# Are the guidelines for coronary artery revascularization, according to the syntax score, being correctly applied, by a heart team?

**DOI:** 10.1186/1749-8090-10-S1-A183

**Published:** 2015-12-16

**Authors:** Rui Almeida, Daniel Almeida, Maurício Centenaro, Alessandro Yassue, Lucas Tonin, John Toigo

**Affiliations:** 1 Faculdade Assis Gurgacz, Cascavel, Paraná, Brazil; 2 Parana Western State University, Cascavel, Paraná, Brazil

## Background/Introduction

The 2014 ESC/EACTS Guidelines on myocardial revascularization, define the SYNTAX Score, as a risk stratification model for the planning the type of treatment. The SYNTAX score was developed to grade the anatomical complexity of coronary lesions in patients with left main or three-vessel disease.

## Aims/Objectives

To assess whether the guidelines, based on SYNTAX Score, are being followed in a cardiovascular unit in the south of Brazil, in which the decision is made by a Heart Team.

## Method

A retrospective blind study was conducted evaluating 395 coronary angiographies, from January 2013 to August 2014. The insertion criteria was the angiography of all patients under investigation for coronary disease. The exclusion criteria were the angiographies of patients that already had any type of percutaneous transluminal angioplasty (PCI) or coronary artery bypass grafting (CABG). The angiographies were evaluated and classified according the SS, in group A (score ≥ 22), B (score 23-32) and C (score ≤ 32). An independent blind observer checked the type of treatment performed and the percentage of each of those three groups.

## Results

The coronary angiographies, of the 395 patients were analyzed and showed an average SS of 9.95 ± 10.45.

The table below shows the population of each group:

**Table 1:** SAMPLE ANALYSIS

Patients	Mean	SS	SD	CL 95
GROUP A	346	2,83	3,23	7,24 ± 0,71
GROUP B	31	11,28	1,12	25,06 ± 2,39
GROUP C	18	22,75	7,89	38,05 ± 3,13

From the 395 patients assigned to the study, 121 patients underwent PCI and 42 CABG. The remainder 232, underwent medical treatment. All patients undergoing CABG were in the C group and part of B; of patients undergoing PCI, 7 (5.79%) belonged to the B group and 114 to group A (94.21%).

## Discussion/Conclusion

The guidelines have been followed, in a unit in which a Heart Team decides what type of treatment should be considered, because the patients underwent the recommended treatment, based on the SYNTAX Score.

